# ExlA: A New Contributor to *Pseudomonas aeruginosa* Virulence

**DOI:** 10.3389/fcimb.2022.929150

**Published:** 2022-06-23

**Authors:** Philippe Huber

**Affiliations:** Université Paris-Saclay, INSERM, CEA, Center for Immunology of Viral, Autoimmune, Hematological and Bacterial Diseases (IMVA-HB/IDMIT), Fontenay-aux-Roses, France

**Keywords:** pore-forming toxins, bacterial virulence factors, animal models of infection, cadherins, inflammasome, two-partner secretion systems

## Abstract

ExlA (also called exolysin) is a recently discovered virulence factor secreted by a subset of *Pseudomonas aeruginosa* strains in which a type 3 secretion system is lacking. *exlA*-positive strains were identified worldwide in the clinic, causing several types of infectious diseases, and were detected in various locations in the environment. ExlA possesses pore-forming activity and is cytolytic for most human cell types. It belongs to a class of poorly characterized bacterial toxins, sharing a similar protein domain organization and a common secretion pathway. This review summarizes the recent findings regarding ExlA synthesis, its secretion pathway, and its toxic behavior for host cells.

## Introduction

A plethora of virulence factors have been described for the opportunistic pathogen *Pseudomonas aeruginosa* (Gellatly and Hancock, 2013; Juan et al., 2017). The most harmful factor is the type 3 secretion system (T3SS) and its substrates, the exoenzymes ExoU, ExoS, ExoT, and ExoY, that are directly injected into the cytoplasm of host cells (Hauser, 2009; Sawa, 2014; Morrow et al., 2017). These toxins trigger cell necrosis (ExoU) or cell rounding caused by actin cytoskeleton breakdown (ExoS and ExoT) or by microtubule disruption (ExoY). The presence of *exoS* or *exoU* in clinical isolates was correlated with high disease severity in several clinical studies or in animal models of *P. aeruginosa* infection (Hauser et al., 2002; Vance et al., 2005; Le Berre et al., 2011; El-Solh et al., 2012).

**Figure 1:**
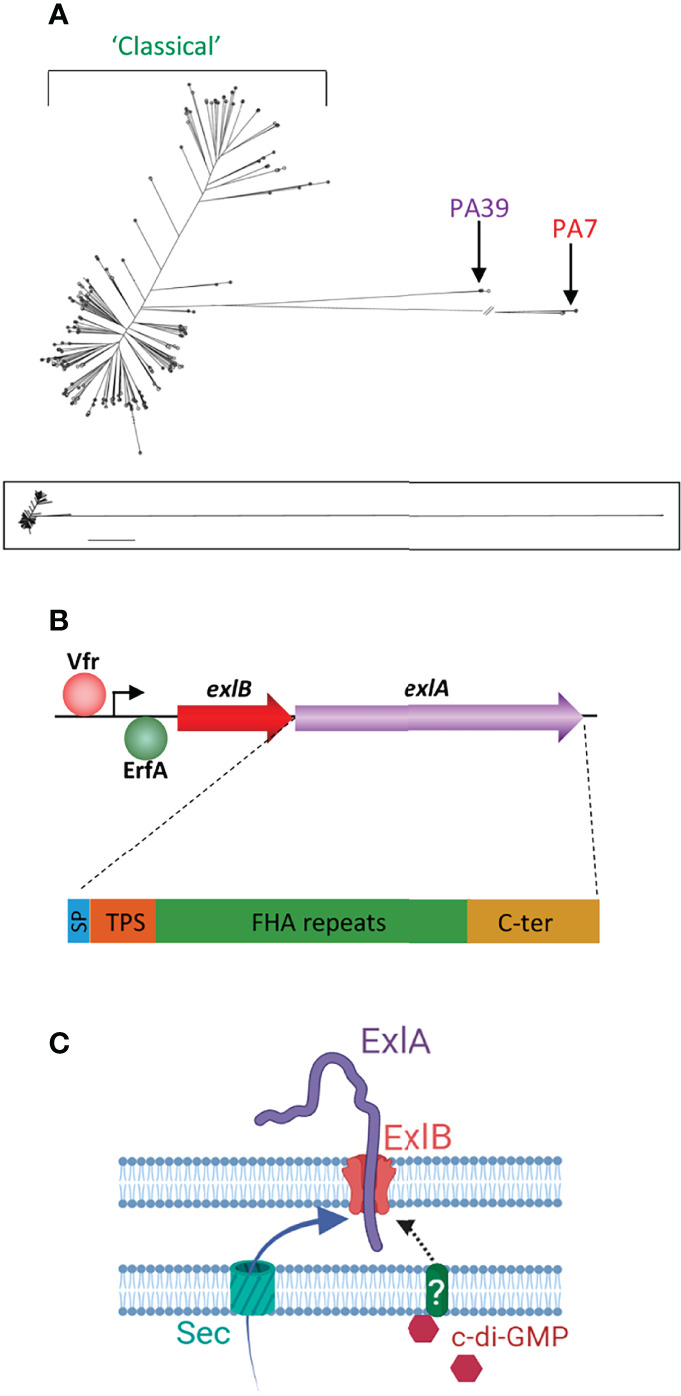
Genetics of the *exlA* strains and the synthesis of ExlA. **(A)** Phylogenetic tree of *Pseudomonas aeruginosa* strains created by genome comparison [derived from Kos et al. (2015)], showing the two genetic outlier clusters, PA7 and PA39. The PA7 strains and most PA39 strains possess the *exlA* gene, as opposed to classical strains. The lower panel shows the entire tree with uninterrupted lines. **(B)** Transcription of the *exlB–exlA* operon is enhanced by Vfr and negatively regulated by the ErfA transcription factor. The 172-kDa ExlA protein consists of a secretion signal peptide (SP), a two-partner secretion (TPS) domain that interacts with ExlB, filamentous hemagglutinin adhesin (FHA)-like repeats, and a C-terminal (C-ter) domain. **(C)** Both ExlA and ExlB are secreted into the periplasm *via* the sec pathway. ExlB is predicted to insert into the outer membrane. ExlA is exported into the extracellular milieu through the pore formed by ExlB. When intracellular concentrations of c-di-GMP are high, ExlA translocation stalls at midway with part of the toxin pointing outside. It is hypothesized that secretion resumes when ExlA interacts with a host receptor. **(C)** Created with BioRender.com.

However, some clinical strains possess neither the genes encoding the exoenzymes nor those for the proteins forming the T3SS. The first described strain of this type was described in 2010 and was called PA7 (Roy et al., 2010). Since then, several PA7-like strains have been identified, and their genomic analysis revealed that they form a group of genetic outliers quite distant from the “classical” strains (**Figure 1A**) (Kos et al., 2015; Reboud et al., 2016; Freschi et al., 2019; Ozer et al., 2019; Sood et al., 2019; Medina-Rojas et al., 2020). Another group emerged, referred to as PA39-like strains, which similarly does not contain the genes for the T3SS and its exoenzymes but is less phylogenetically different from the classical strains (**Figure 1A**) (Dingemans et al., 2014).

In 2014, Elsen et al. reported that a PA7-like strain called CLJ1, isolated from a patient with hemorrhagic pneumonia, possessed a novel virulence factor named exolysin or ExlA, which is mainly responsible for the toxic behavior of this highly virulent strain (Elsen et al., 2014). ExlA belongs to the ExlAB two-partner secretion system (TPS) in which ExlA is the passenger protein and ExlB the transporter, presumably located in the bacterial outer membrane. ExlA is a secreted protein exhibiting pore-forming activities in eukaryotic cells. The genes encoding the ExlAB TPS have been identified in all tested PA7-like and most PA39-like strains (Reboud et al., 2016; Medina-Rojas et al., 2020; Ruiz-Roldan et al., 2020). Interestingly, genes coding for ExlA-like proteins have been found in other *Pseudomonas* species (*P. putida*, *P. entomophila*, and *P. protegens*) (Basso et al., 2017a). ExlA is the only pore-forming toxin described in *P. aeruginosa*, aside from the T3SS translocon inserted into the plasma membrane of target cells.

This review recapitulates the main findings on ExlA biology from the transcription of its gene to its action on cells, animals, and man.

## Biochemical Characteristics

ExlA is a 172-kDa protein composed of three functional domains, in addition to an N-terminal type I secretion signal peptide (SP) (**Figure 1B**) (Basso et al., 2017a). A conserved TPS secretion domain, required for interaction with ExlB POTRA (for *po*lypeptide-associated *tra*nsport) domains, is located downstream of SP. The large central domain consists of sequence repeats homologous to *Bordetella pertussis* TPS FHA (*f*ilamentous *h*emagglutinin *a*dhesin), which is predicted to fold into an elongated β-helix. The C-terminal domain, which usually confers to TPS passengers their specificity of action, is unrelated to other domains with known functions and is shown to form a molten globule in solution (Bertrand et al., 2020).

The ExlA domain organization and secretion pathway are features shared by other bacterial toxins exhibiting cytolytic activities, including ShlA in *Serratia marcescens*, HpmA in *Proteus mirabilis*, EthA in *Edwardsiella tarda*, and HhdA in *Haemophilus ducreyi* (Elsen et al., 2014; Guerin et al., 2017). These proteins form a family of toxins, whose action modes are poorly characterized, as opposed to most known bacterial pore-forming toxins (Dal Peraro and van der Goot, 2016).

## ExlA Genetics and Transcriptional Regulation

The ExlAB TPS is encoded by the *exlB–exlA* operon (**Figure 1B**). The promoter region contains a binding site for Vfr (Berry et al., 2018), a transcription factor known to regulate several virulence factors of *P. aeruginosa* (Fuchs et al., 2010). Vfr upregulates *exlB–exlA* expression leading to increased ExlA synthesis, and its binding to the *exlB–exlA* promoter is cAMP-dependent. The membrane-bound adenylate cyclase CyaB—and not the cytosolic adenylate cyclase CyaA—is responsible for cAMP-dependent *exlB–exlA* transcriptional activation, suggesting that *exlB*–*exlA* expression is upregulated by some environmental cues, such as low calcium concentrations or attachment to surfaces (Berry et al., 2018). The upregulation effect was confirmed when bacteria were grown in low calcium concentrations; this effect has, however, not been investigated for surface attachment.

In addition, *exlB–exlA* transcription is repressed by a very potent transcriptional factor named ErfA, for *exlA*-*r*epression *f*actor A, which binds to a motif located in the *exlB–exlA* 5′-untranslated region. *erfA* inactivation leads to an ~40-fold induction of the operon expression and to a dramatic increase in strain virulence (Trouillon et al., 2020). Interestingly, ErfA is present in all tested *P. aeruginosa* strains and is shown to regulate several other genes, unrelated to virulence factors.

## ExlA Secretion

Both ExlA and ExlB are predicted to be secreted into the periplasm by the sec pathway. ExlA accumulates at the outer membrane as shown by cellular fractionation experiments (Deruelle et al., 2021) (**Figure 1C**). Microscopy immunolocalization identified ExlA spots at the bacterial periphery. However, spots were only present in 0.4% of bacteria, suggesting that only a very small proportion of bacteria actively secrete ExlA. In these bacteria, only one spot per cell was detected that could be positioned in any location (along the sides or at the poles) of the bacterial membrane. No spots were identified in the *exlB* mutant bacteria, indicating that ExlA is associated with ExlB at the membrane in the wild-type strain. In *erfA* mutant bacteria, the proportion of ExlA-positive cells increased to ~10%, confirming that ExlAB synthesis repression is induced by ErfA. In the *erfA* mutant, the signal intensities increased rather than the number of spots, suggesting that multiple ExlB proteins aggregate in the same membrane location when they are overproduced (Deruelle et al., 2021).

Only very low amounts of ExlA are detected in PA7-like strain secretomes (Reboud et al., 2016), and they are at the limit of detection by ELISA (Deruelle et al., 2021). Furthermore, secretomes do not induce the cytolytic activity observed when cells are in direct contact with bacteria, leading to the hypothesis that ExlA toxicity is contact-dependent (Basso et al., 2017a). This is further suggested by the lack of toxicity of isogenic mutants deficient in type 4 pili, which are known to promote the interaction of bacteria with host cells (Basso et al., 2017a). The current hypothesis is thus that the active form of the toxin is that anchored by ExlB in the outer membrane, rather than the secreted protein (**Figure 1C**). Proteinase digestion experiments showed that a part of ExlA is pointing out of the bacterial membrane and further supports a model in which ExlA, entrapped in the outer membrane, could interact with a potential receptor located at the host cell surface (Deruelle et al., 2021). This secretion model is analogous to that described for contact-dependent inhibition (CDI) toxins, belonging to the TPS family, for which passenger translocation across the outer membrane stalls at mid-length in the TPS transporter and resumes upon interaction with a target receptor (Ruhe et al., 2018). Other examples of TPS in which the passenger is entrapped into the transporter include the *Haemophilus influenzae* adhesin HMW1 (Grass et al., 2015) and *P. aeruginosa* CdrA, a protein known to mediate cell–cell aggregation and biofilm maturation (Cooley et al., 2016).

ExlA secretion from the outer membrane into the extracellular milieu is controlled by cyclic-di-GMP (**Figure 1C**), a second messenger known to induce the production of biofilm components by *P. aeruginosa* (Hall and Lee, 2018). High bacterial concentrations of c-di-GMP have no effect on *exlB–exlA* transcription but retain ExlA at the membrane, which is, as previously mentioned, predicted to increase bacterial virulence (Deruelle et al., 2021). The signal transduction pathway transferring the signal from cytosolic c-di-GMP to ExlA and ExlB, both located at the outer membrane, remains unknown and probably involves shuttle proteins in the periplasm.

Hence, ExlA delivery is tightly controlled at the transcriptional and secretion levels by two second messengers that are both upregulated by surface interaction with type 4 pili (Luo et al., 2015). Thus, the lower virulence of pili-deficient strains might also be caused by the lack of cAMP and c-di-GMP induction (when bacteria are close to the host cells), in addition to their inability to “present” ExlA to its cognate eukaryotic receptor.

## ExlA Insertion Into the Host Cell Membrane

Most bacterial pore-forming toxins interact with a proteinaceous or lipidic receptor located at the host plasma membrane (Dal Peraro and van der Goot, 2016). No eukaryotic receptor has been reported for ExlA or ExlA-related toxins (ShlA, HpmA, EthA, and HhdA). Studies were hampered by the rapid degradation of these toxins in solution. *exlA*-positive bacteria are highly cytolytic for all tested cell types (epithelial, endothelial, fibroblastic, and immune cells, although poorly for erythrocytes) (Reboud et al., 2016), suggesting that the ExlA receptor, if any, might be relatively largely distributed in human cells.

ExlA inserts into the lipid raft domain of the host plasma membrane, where it seems highly stable, as no degradation bands were observed by electrophoresis (Bertrand et al., 2020). Whether ExlA oligomerizes and if the process occurs at this stage—like other pore-forming toxins—are unknown.

Experiments with osmoprotectants of different sizes demonstrated that ExlA induces the formation of a 1.6-nm diameter pore (Basso et al., 2017a). The C-terminal domain is required for pore formation in cells and is sufficient to produce membrane punctures in artificial membranes (Bertrand et al., 2020). However, the N-terminal part of the toxin (deleted from the C-terminal domain), which similarly inserts into lipid rafts, is also able to permeabilize the host membrane and induce cytolysis, yet with slower kinetics (Bertrand et al., 2020). The cytolytic effect is, however, not perturbed by the presence of osmoprotectants, suggesting that the N-terminal domain does not form a pore *per se* (Basso et al., 2017a). ExlA may thus possess two cytolytic activities localized in two parts of the protein and operating with different mechanisms. The synergistic effect of the two domains is not documented.

## ExlA Toxicity in Epithelial and Endothelial Cells

The first sign of pore formation is the massive entry of calcium that can be detected by calcium imaging (Reboud et al., 2017). This is caused by the important calcium concentration gradient between the extracellular milieu and the cytosol.

A sustained increase in intracellular calcium concentrations is poisonous for cells (Bouillot et al., 2018), and ExlA-dependent calcium influx has several devastating consequences (Reboud et al., 2017).

**Figure 2:**
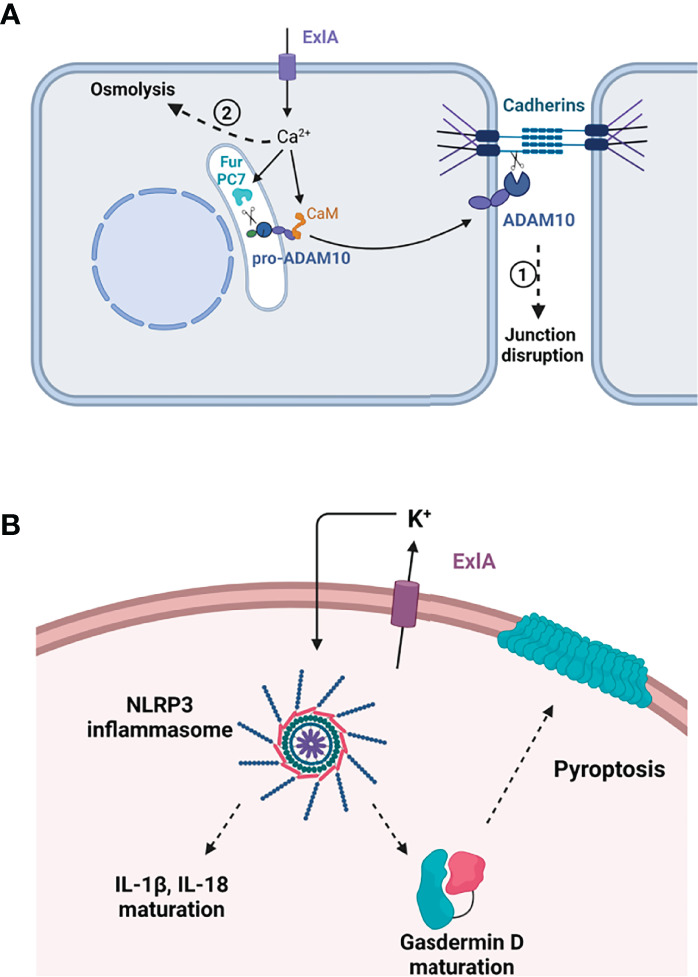
Effect of ExlA pore formation in host cells. **(A)** Effect in epithelial and endothelial cells. Pore formation induces massive Ca2+ influx, which activates two processes. In the first process, Ca2+ binds to calmodulin (CaM), releasing calmodulin from pro-ADAM10, and activates two convertases, furin (Fur) and PC7, which excise the ADAM10 prodomain. Mature ADAM10 is then exported to the plasma membrane where it cleaves the cadherin ectodomain, leading to junction disruption. In the second phase, cells die from osmolysis owing to ion imbalance. **(B)** In macrophages, ExlA induces cell death by pyroptosis. K+ efflux through ExlA pores induces the assembly of the NLRP3 inflammasome, thereby activating caspase-1. Caspase-1 excises IL-1β and IL-18 prodomains and cleaves pro-gasdermin D. Mature gasdermin D oligomerizes and oligomers form a large pore in the plasma membrane, allowing the release of inflammatory cytokines. **(A, B)** Created with BioRender.com.

The first calcium-dependent effect that can be noted is cell rounding and cell–cell junction disruption (**Figure 2A**). Adherens junctions are mainly formed by the homophilic interaction between cadherin molecules localized at the border of adjacent cells (Garcia et al., 2018). E-cadherin is present at epithelial junctions and VE-cadherin at endothelial junctions, and both engage in homotypic interactions. Cadherin adhesion is regulated by multiple transduction pathways that can lead to reversible or irreversible modifications in the protein. One irreversible modification is the shedding of the entire cadherin ectodomain by the transmembrane protease ADAM10 (Maretzky et al., 2005; Dreymueller et al., 2012). When cells are incubated with PA7-like bacteria, E- or VE-cadherin ectodomains are shed in an ExlA- and ADAM10-dependent manner (Reboud et al., 2017). ADAM10 prodomain endoproteolysis is required to unmask its catalytic domain. ADAM10 maturation from its precursor protein is regulated by calcium at two levels: i) its N-terminal prodomain is cleaved by proprotein convertases, either PC7 or furin, whose proteolytic activities are calcium-dependent (Anders et al., 2001); and ii) calmodulin, a protein with high affinity for calcium, binds to ADAM10 in the absence of calcium (Nagano et al., 2004; Ponnuchamy and Khalil, 2008). When calmodulin interacts with calcium, it releases ADAM10, which becomes available for endoproteolysis and export. Hence, by lifting the calmodulin inhibition and enhancing PC7 and furin activities, the ExlA-induced calcium rise is a trigger for ADAM10 maturation and export to the plasma membrane, where it can cleave the cadherins and dismantle the adherens junctions. Activation of the calcium–ADAM10–cadherin axis was also described for other pore-forming toxins: one of the same class, ShlA (Reboud et al., 2017), and two others, HlA and PLY, from *Staphylococcus aureus* and *Streptococcus pneumoniae*, respectively (Inoshima et al., 2011). It is thus likely a common effect of calcium-permissive pores.

Later on, usually within 1.5–3 h, the infected cells are subjected to osmolysis by a process also involving intracellular calcium influx, as loading cells with the calcium quencher BAPTA-AM delay cell burst (Reboud et al., 2017). As previously mentioned, calcium has deleterious effects on various cellular components, including the mitochondria and endoplasmic reticulum, and several signaling pathways [reviewed in Bouillot et al. (2018)], and can cause osmolysis in itself. Whether other death pathways independent of calcium influx are activated by *exlA*-positive bacteria in epithelial or endothelial cells remains elusive.

Importantly, ExlA pores are not eliminated by host cells, as opposed to some other pore-forming toxins, which can be excluded by microvesicle externalization or by endocytosis (Bouillot et al., 2018). For ExlA, all cells showing calcium influx inevitably die after a period of 2–3 h, indicating that ExlA pores cannot be excluded (Reboud et al., 2017), a property shared by toxins forming small pores (diameter < 2.5 nm).

## Inflammasome Activation by ExlA

ExlA was shown to induce another deadly process in macrophages called “pyroptosis,” as previously shown for multiple pore-forming toxins (Basso et al., 2017b). By allowing potassium efflux, pore-forming toxins trigger the assembly of the NLRP3 inflammasome in immune cells, which activates caspase-1 protease (Storek and Monack, 2015). This activation has two major effects: i) maturation of IL-1β and IL-18, two “initiators” of the inflammatory cytokine response, whose synthesis is enhanced by TLR (*T*oll-*l*ike *r*eceptor) signaling; and ii) maturation of gasdermin D, a pore-forming protein from the host, which provokes cell death by osmolysis, thereby releasing mature IL-1β and IL-18 into the extracellular compartment (**Figure 2B**). The overall process has a major impact on the recruitment and activation of immune cells at the site of infection.

## Pathogeny of PA7-Like Strains in Man and Animal Models

*exlA*-positive clinical strains have been isolated from patients with chronic (notably suffering from cystic fibrosis or chronic obstructive pulmonary disease) or acute infectious diseases at different localizations: mostly in the lung, blood, and urinary tract, but also in wounds, peritoneum, and ear (Reboud et al., 2016; Medina-Rojas et al., 2020). Three systematic genome analyses of *P. aeruginosa* clinical isolates revealed that PA7-like strains might represent 1.5%–2% of the total *P. aeruginosa* infections (Freschi et al., 2019; Ozer et al., 2019; Medina-Rojas et al., 2020), which is low with regard to the success of their worldwide distribution.

These strains have also been identified in the environment, such as on a plant, in the soil, and in wild-animal feces, which demonstrates their capacity to adapt and thrive in various environmental types (Reboud et al., 2016; Ruiz-Roldan et al., 2020).

The toxicity levels of *exlA* strains on human cells *in vitro* are directly correlated with the amounts of synthesized ExlA (Reboud et al., 2016). With the exception of the hypervirulent CLJ1 strain, *exlA*-positive strains are overall less toxic *in vivo* than the T3SS-positive strains, both in the mouse pneumonia and *Galleria mellonella* infection models, but are still lethal (Reboud et al., 2016; Medina-Rojas et al., 2020).

Their toxicity in the mouse pneumonia model also depends upon the extent of the immune response they trigger, and this effect greatly varies between strains. This feature was further documented by the analysis of the virulence potential of two strains, CLJ1 and IHMA87, with similar toxicity levels on cells but with different abilities to induce an immune response *in vivo*. CLJ1 is devoid of type 4 pili and flagellum, two major bacterial determinants inducing TLR signaling, which culminates in inflammatory cytokine gene upregulation. CLJ1 proliferated in the mouse lung, inducing major alveolar lesions and hemorrhages, which confirms its high necrotizing potential (Bouillot et al., 2017). The survival curve of CLJ1-infected mice was similar to that of mice infected with a highly virulent ExoU-secreting strain.

Similar experiments were performed with IHMA87, which harbors type 4 pili and a flagellum that can both activate TLR signaling. As opposed to CLJ1, lung bacterial burdens decreased within days following infection with IHMA87, and histological lesions were less dramatic (Bouillot et al., 2020). IHMA87 led to less deadly infections than CLJ1, and part of its virulence could be attributed to inflammation exacerbation, as IHMA87-infected mice deficient in inflammatory caspases were less prone to fatal infections.

Thus, ExlA toxicity may be tempered by other bacterial determinants facilitating bacteria suppression by the host immune system. Nevertheless, *exlA*-positive bacteria are generally more resistant to antibiotics than the classical strains, which may provide a selection advantage to these genetic outliers (Medina-Rojas et al., 2020).

## Conclusion and Future Prospects

Although several mechanisms of ExlA synthesis and action on host cells have been uncovered in recent years, several important questions remain with regard to ExlA insertion into the host membranes: the identification of one or more receptors at the host surface, the convergent contribution of the N- and C-terminal domains in membrane permeability, the possible oligomerization of the toxin, and a comprehensive scenario of pore formation and insertion are all critical issues that need to be tackled.

As previously mentioned, very little information is available on the action mechanisms of ExlA-related toxins, except for ShlA, whose biochemistry and toxicity have been investigated by a few groups since the 1980s. In particular, Braun’s lab showed that ShlA was modified by its secretion through ShlB (Walker et al., 2004). It is unclear at present if ExlA passage through ExlB modifies its structure and activity.

More generally, it will be important to determine if common rules apply to this class of pore-forming toxins, and any property identified in one of these toxins should be assessed in at least one other toxin of this class.

Finally, in the coming years, it will be important to track the onset of new *exlA*-positive clinical isolates to see whether they have a selective advantage compared to the classical strains because of their higher antibioresistance and lower toxicity.

## Author Contributions

PH wrote the manuscript.

## Funding

This work was funded by institutional grants from the CEA and INSERM.

## Conflict of Interest

The author declares that the research was conducted in the absence of any commercial or financial relationships that could be construed as a potential conflict of interest.

## Publisher’s Note

All claims expressed in this article are solely those of the authors and do not necessarily represent those of their affiliated organizations, or those of the publisher, the editors and the reviewers. Any product that may be evaluated in this article, or claim that may be made by its manufacturer, is not guaranteed or endorsed by the publisher.
